# Ankylostomiasis in Cornwall

**Published:** 1903-10-17

**Authors:** 


### Ankylostomiasis in Cornwall.

The appearance of Ankylostomiasis in a Cornish mine at Dolcoath, near Camborne, is an event which may well excite grave anxiety among the members of the great industry which would chiefly be imperilled by its prevalence; and it is therefore eminently satisfactory that not only has the attention of the Home Office been directed towards the natural history of the disease, and towards the methods by which its extension may be controlled, but also that the miners themselves, as represented at their conference which has just been held at Glasgow, have listened with close interest to a lecture by Dr. Court, in which the most important facts of the case were set forth with lucidity and vigour, and appear to have made a profound impression upon the minds of the audience. The parasitic nematode worm, *Ankylostoma duodenale*, by which the disease is caused, and from which it derives its somewhat appalling designation, was first discovered by Griesinger in the intestines of patients suffering from what was then known as Egyptian chlorosis, and was afterwards identified by Wucherer as the cause of an analogous condition of frequent occurrence in Brazil. Attention having once been directed to the subject, the parasite was soon discovered in other localities, and was found before long to be the source of a very common, widespread, and troublesome malady in tropical and sub-tropical countries all over the world. Its first recorded appearance in Europe was among the men engaged in the construction of the St. Gothard Tunnel, many of whom died from the effects of what was at first described as "miners' anæmia," but which was shown by Perroncito to be ankylostomiasis. It was soon carried from the Alps to the mines of Austria and Hungary, in some of which, before 1896, some 70 per cent. of the workers are said to have been affected. It appeared also in the coal mines at St. Etienne, and in Belgium, and in 1896 it invaded the great German coal district in Westphalia, producing an annual average of about 100 cases until 1890. In that year a system of free watering was introduced into the Westphalian mines as a protection against explosions, and against the consequent firing of dust, and the disease immediately increased in prevalence and in severity. More than 1,000 cases were reported in 1901, and more than 1,300 in the first nine months of 1902, since which time the increase is said to have been still more rapid than before. A few cases had been identified in this country by Sir Patrick Manson, among Lascars in vessels from India, but the disease was not detected in Englishmen until last year, when the amount of illness among certain miners in Cornwall led to inquiry by the Home Office, and Dr. Haldane, F.R.S., was sent down to investigate the conditions. The cases were originally described as "anæmia," and were attributed to defective ventilation; but Dr. Haldane found the ventilation excellent, and was not long in discovering the parasite.

The *Ankylostoma duodenale* is a tiny worm, not more than half an inch long, which is found in large numbers in the upper portions of the intestinal canal, where it attaches itself to the mucous membrane, and where it liberates great multitudes of ova, which mingle with the contents of the intestine and are evacuated with the fæces. The worm occasions great depravation of the quality of the blood, and the consequent anæmia is the first indication of its presence. The patients become pallid, suffer from palpitations and difficulty of breathing, cannot mount ladders without risk of syncope, and are generally incapacitated from exertion. In the meanwhile the patient discharges innumerable ova from the bowels, and these ova, in the ordinary conditions of a mine, become very generally diffused among the mud of the gangways, are carried here and there by the boots of the men, and are presumably swallowed with the food that is habitually taken with unwashen hands. Once swallowed they undergo development in the intestines of the host, and so the circle is continued. The treatment of actual cases resolves itself into removal of the patient from conditions contributory to re-infection, and into destruction of the parasites by suitable medicines, among which the extract of male fern is preferred in Westphalia, and thymol by Dr. Haldane. The completeness of the cure can only be ascertained by microscopic examination of the fæces for worms or ova, and, as long as either of these are voided, the treatment must be continued. More important than the treatment, however, is prevention; and this can only be secured by the exercise of proper control over the evacuations, or, in other words, by a strict system of mine discipline with regard to them which can only be enforced by the men themselves. All necessary facilities may be provided by employers; but the case, even then, is like that of taking a horse to the water. It is for this reason that especial value attaches to such lectures as the one recently given by Dr. Court; since it is only by making a somewhat reckless set of men understand the sources of danger that they can be expected to lend their assistance in avoiding them. In relation to this part of the subject, Dr. Court cautioned his hearers against the admission of foreign workmen, unable to understand the rules laid down for their guidance, unless they were first ascertained to be free from parasites.

